# Distinct compartmentalization of SP-A and SP-D in the vasculature and lungs of patients with idiopathic pulmonary fibrosis

**DOI:** 10.1186/1471-2466-14-196

**Published:** 2014-12-08

**Authors:** Hirotaka Nishikiori, Hirofumi Chiba, Shigeru Ariki, Koji Kuronuma, Mitsuo Otsuka, Masanori Shiratori, Kimiyuki Ikeda, Atsushi Watanabe, Yoshio Kuroki, Hiroki Takahashi

**Affiliations:** Department of Respiratory Medicine and Allergology, Sapporo Medical University School of Medicine, South-1 West-17, Chuo-ku, Sapporo, 060-8556 Japan; Department of Biochemistry, Sapporo Medical University School of Medicine, Sapporo, Japan; Department of Thoracic Surgery, Sapporo Medical University School of Medicine, Sapporo, Japan

**Keywords:** Surfactant protein, SP-A, SP-D, Idiopathic pulmonary fibrosis, Bronchoalveolar lavage, Biomarker, Hydrophilicity, Immunohistochemistry, Compartmentalization

## Abstract

**Background:**

Surfactant proteins SP-A and SP-D are useful biomarkers in diagnosis, monitoring, and prognosis of idiopathic pulmonary fibrosis (IPF). Despite their high structural homology, their serum concentrations often vary in IPF patients. This retrospective study aimed to investigate distinct compartmentalization of SP-A and SP-D in the vasculature and lungs by bronchoalveolar lavage fluid (BALF)/serum analysis, hydrophilicity and immunohistochemistry.

**Methods:**

We included 36 IPF patients, 18 sarcoidosis (SAR) patients and 20 healthy subjects. Low-speed centrifugal supernatants of BALF (Sup-1) were obtained from each subject. Sera were also collected from each patient. Furthermore, we separated Sup-1 of IPF patients into hydrophilic supernatant (Sup-2) and hydrophobic precipitate (Ppt) by high-speed centrifugation. We measured SP-A and SP-D levels of each sample with the sandwich ELISA technique. We analyzed the change of the BALF/serum level ratios of the two proteins in IPF patients and their hydrophilicity in BALF. The distribution in the IPF lungs was also examined by immunohistochemical staining.

**Results:**

In BALF, SP-A levels were comparable between the groups; however, SP-D levels were significantly lower in IPF patients than in others. Although IPF reduced the BALF/serum level ratios of the two proteins, the change in concentration of SP-D was more evident than SP-A. This suggests a higher disease impact for SP-D. Regarding hydrophilicity, although more than half of the SP-D remained in hydrophilic fractions (Sup-2), almost all of the SP-A sedimented in the Ppt with phospholipids. Hydrophilicity suggests that SP-D migrates into the blood more easily than SP-A in IPF lungs. Immunohistochemistry revealed that SP-A was confined to thick mucus-filling alveolar space, whereas SP-D was often intravascular. This data also suggests that SP-D easily leaks into the bloodstream, whereas SP-A remains bound to surfactant lipids in the alveolar space.

**Conclusions:**

The current study investigated distinct compartmentalization of SP-A and SP-D in the vasculature and lungs. Our results suggest that serum levels of SP-D could reflect pathological changes of the IPF lungs more incisively than those of SP-A.

## Background

The pulmonary surfactant consists of phospholipids, mainly dipalmitoyl phosphatidylcholine (DPPC), surfactant protein (SP)-A, SP-B, SP-C, and SP-D. Both SP-A and SP-D are produced by type II alveolar epithelial cells and Clara cells, and are secreted into the alveolar space [1, 2]. Unlike SP-B and SP-C, they are hydrophilic proteins belonging to the C-type lectin superfamily. Both SP-A and SP-D play crucial roles in lung innate immunity [3, 4]. Moreover, their serum levels reflect the severity and prognosis of interstitial lung diseases, including idiopathic pulmonary fibrosis (IPF) [5, 6]. Although SP-A and SP-D are very similar in terms of structures and functions [7, 8], they have different binding specificities for phospholipids or glycolipids. Whereas SP-A specifically binds to DPPC and galactosylceramide, SP-D binds to phosphatidylinositol and glucosylceramide [9–12].

Patients diagnosed with IPF have a poor prognosis (median survival approximately 3 years), with considerable individual variability in the clinical course [13, 14]. Therefore, it is important to predict each patient’s prognosis correctly for treatment optimization. Several prognostic factors of IPF have been proposed, including age, forced vital capacity (FVC), diffusing capacity (D_LCO_), chronological changes in respiratory functions, and desaturation during the 6-min walk test [15–18]. On the other hand, blood biomarkers of IPF are preferred because they do not require an effort from the patients and are easy to measure, namely SP-A, SP-D, Krebs von den Lungen-6 (KL-6), matrix metalloproteinase-7 (MMP-7), and chemokine (C-C motif) ligand 18 (CCL18) [5, 6, 19–22]. In Japan, SP-A, SP-D, and KL-6 are the most commonly used serum biomarkers in diagnosis, monitoring, and prognosis of IPF, with empirical data having accumulated for over 10 years.

Although SP-A and SP-D are proteins with high homology, we often experience IPF cases with differences in serum levels for the two proteins [5]. In this study, we investigated dissociation between SP-A and SP-D in terms of hydrophilicity, their levels in serum and bronchoalveolar lavage fluid (BALF), and tissue distribution by immunohistochemistry in healthy and IPF lungs.

## Methods

### Study subjects

This retrospective study included 54 consecutive patients [36 IPF; 18 sarcoidosis (SAR)] who had a bronchoalveolar lavage (BAL) performed at the Sapporo Medical University Hospital from December 2003 to May 2012. SAR patients were used as disease controls because, in Japan, they seldom show fibrotic changes in the lung, even with pulmonary lesions [23, 24]. The diagnosis of IPF was based on pathological, clinical, and radiological findings, according to the 2010 American Thoracic Society (ATS)/European Respiratory Society (ERS)/Japanese Respiratory Society (JRS)/Latin American Thoracic Association (ALAT) statement [25]. A surgical lung biopsy was performed on 12 IPF patients. Sarcoidosis was diagnosed clinically (N = 18) and histopathologically (N = 11) using the 2006 Japanese diagnostic standard and guidelines for sarcoidosis. The Japanese guideline specifies the following diagnostic criteria. 1) Histological demonstration of non-caseating epithelioid cell granuloma in one organ in addition to one or more of the following conditions: a) the same granuloma in other organs; b) typical clinical findings in other organs (chest radiographic findings, bronchoscopic findings, typical ocular symptoms, cardiac symptoms and/or findings, typical skin eruption, and neuropathic and/or myopathic symptoms); c) two or more of the following abnormalities on examinations that reflect a systemic response of sarcoidosis: bilateral hilar lymphadenopathy, elevated serum angiotensin-converting enzyme levels, negative tuberculin skin test, abnormal localizations on gallium scintigraphy, elevated lymphocytes and/or a high CD4/CD8 ratio in BALF, and hypercalcemia and/or hypercalciuria. 2) In patients with no histological evidence, the above-mentioned typical clinical findings in two or more organs in addition to abnormal results in two or more of the above-mentioned six examinations. 3) Exclusion of other diseases showing similar histological or clinical findings. One patient was diagnosed by surgical lung biopsy, seven by transbronchial lung biopsy, and three by biopsy of other organs. Some patients had granular shadows or nodules on their lung field, but no fibrotic pattern. At the time of BAL fluid collection, none of the patients received corticosteroids or immunosuppressants and had any infection or any nutrition problems.

To compare BALF concentration of the two proteins, this study also included a control group consisting of 20 healthy subjects from our previous study in which BAL was conducted under the same conditions [26]. They had no history of lung disease, and there was no abnormal finding on physical examination, chest radiograph, and pulmonary function tests. This research was conducted in accordance with the Declaration of Helsinki (1964), and has obtained the approval of the Ethics Committee of the Sapporo Medical University.

### BAL technique

Each participant received an intramuscular injection of atropine sulfate and hydroxyzine or pethidine, followed by local anesthesia with lidocaine. A flexible bronchofiberscope (OLYMPUS, Tokyo, Japan) was inserted orally to pour 50 mL of 0.9% saline (37°C) into the bronchus of the right middle lobe or the left lingula, and the BALF was recovered. This process was repeated three times and the fractions pooled together. BALF was centrifuged (5 min; 1,000 *g*) to remove cell components, and this Sup-1 was cryopreserved at -80°C until use.

### Measurements of SP-A and SP-D levels

The SP-A content of Sup-1 was measured using a sandwich enzyme linked immunosorbent assay (ELISA), named SP-A Test/Kokusai-F (Sysmex Co., Kobe, Japan), as previously described [27]. A standard ELISA kit was used to measure SP-D (Yamasa Co. Choshi, Japan), including recombinant human SP-D as the standard, and two monoclonal antibodies against human SP-D (6B2 and 7C6). In addition, peripheral venous blood samples were obtained from each patient within 1 week before BAL collection. Serum SP-A and SP-D levels were determined using the same techniques. We calculated BALF/serum level ratios of the two proteins for each patient, and compared the ratios of two diseases.

### Hydrophilicity of SP-A and SP-D

The hydrophilicity of SP-A and SP-D in the BALF of IPF patients (N = 19) were measured after centrifugation of 1.2 mL of Sup-1 (33,000 *g*; 4°C; 16 h) into a supernatant (Sup-2) and precipitate (Ppt). The Ppt was resuspended in 1.2 mL of 0.9% saline. The concentrations of SP-A and SP-D in Sup-2 and Ppt were measured by ELISA as stated above. Following which, the phospholipids were extracted from Sup-2 and Ppt by the method of Bligh and Dyer [28], and the total amount was measured by the method of Bartlett [29].

### Immunohistochemistry

Immunohistochemical analysis was performed on surgical lung biopsies from 12 IPF patients, as well as peripheral noncancerous control lung tissue from three patients with primary lung cancer obtained by lobectomy. The tissue samples were fixed in 10% buffered formalin (18 h) and embedded in paraffin. Then, 3-μm thick sections were deparaffinized and stained using the following protocols. The primary antibodies were mouse anti-human SP-A monoclonal antibodies (PE10, IBL, JAPAN), and mouse anti-human SP-D monoclonal antibodies (12G5, Abcam, UK). Thereafter, the sections were incubated with biotin-labeled secondary antibody (VECTASTAIN ABC Mouse IgG Kit; Vector Labs, USA), and stained using the DAB Peroxidase Substrate Kit (Vector Labs, USA). For SP-D, samples were pretreated in 10 mM citric acid solution (121°C; 15 min) before being incubated with the primary antibody.

Furthermore, the stained sections were boiled in 10 mM citric acid solution (121°C; 15 min) to remove antibodies, and stained again with monoclonal CD34 (NU-4A1, Nichirei, JAPAN) or monoclonal D2-40 (N1607, Dako, USA) as primary antibodies. Double immunostaining was performed using the Vectastain ABC-AP Mouse IgG Kit (Vector Labs, USA) and the alkaline phosphatase substrate kit (Vector Red; Vector Labs, USA). CD34 stains vascular endothelium, particularly in small capillaries [30], whereas D2-40 stains lymphatic endothelium [31].

The semi-qualitative analysis of immunohistochemistry was performed with concordance of three well-trained pulmonologists who had knowledge of the pathologic features of interstitial pulmonary diseases. We defined immunohistochemical intensities of normal type II epithelial cells as positive; “+”, and expressed those of each area using five grades: “-”, none; “±”, more or less; “+”, positive; “++”, more intense; and “+++”, very intense.

### Statistical analysis

All analyses were performed using the SPSS Statistics 21 (IBM Inc.). The data of age and the BALF recovery rate with symmetric distribution are expressed as mean ± standard deviation, and the differences between the three groups were tested using one-way ANOVA. The differences between each pair of the three groups were tested using Tukey’s HSD test. The data of SP-A and SP-D levels in the serum and BALF, the BALF/serum level ratios of the two proteins, and the percentages of surfactant proteins and phospholipids fragmentation in Sup-2 or Ppt showed asymmetric distributions. These data are expressed as median (interquartile range), and tested using the Mann–Whitney *U*–test (between the two groups) or the Kruskal Wallis test (among three groups). The differences between each pair of the three groups were tested using the Mann–Whitney *U*–test with Bonferroni correction. The differences in sex and the smoking status were tested using the chi-square test. The correlations between the two variables were evaluated using the Spearman’s rank correlation coefficient. All P values < 0.05 were considered statistically significant.

## Results

### Baseline patient characteristics

The basic characteristics and recovery rates of BALF of each subject group are summarized in Table 1. Most IPF patients and healthy subjects were men, whereas both genders were almost equally represented in the SAR patients. The mean age of both the disease groups was 2-fold higher than that of the healthy group (P < 0.001). The smoking status of disease groups was also different from that of the healthy group. Moreover, the percent recovery of BALF was significantly lower in both the disease groups, compared with the healthy group.

**Table 1:** **Patient characteristics and the data of serum and BALF levels of SP-A and SP-D**

	IPF	SAR	HS	
N	36	18	20	
Sex (male: female)	18:8	8:10 P = 0.073	17:3 P = 0.339	P = 0.004
Age (years old)	67.6 ± 8.0	53.7 ± 16.2 P < 0.001	28.2 ± 6.3 P < 0.001	P < 0.001
Smoking status (current: ex-: never smoker)	7: 20: 9	4: 8: 6 P = 0.528	8: 0: 12 P < 0.001	P < 0.001
BALF recovery (%)	51.7 ± 16.5	57.9 ± 12.5 P = 0.513	66.1 ± 22.4 P = 0.001	P = 0.002
Serum SP-A level (ng/ml)	84.5 (55.5 – 115.8)	33.3 (26.1 – 52.0) P < 0.001	-	
Serum SP-D level (ng/ml)	272.0 (172.0 – 441.8)	75.0 (48.8 – 97.3) P < 0.001	-	
BALF SP-A level (ng/ml)	1275.0 (581.0 – 2672.5)	1600.0 (972.0 – 3220.0)	1978.0 (1380.0 – 3749.0)	P = 0.245
BALF SP-D level (ng/ml)	379.0 (223.8 – 570.8)	704.5 (389.3 – 967.5) P = 0.003	667.0 (391.0 – 1219.0) P = 0.003	P < 0.001
BALF/serum ratio of SP-A levels	19.1 (6.9 – 41.6)	51.2 (18.5 – 103.8) P = 0.01	-	
BALF/serum ratio of SP-D levels	1.3 (0.7 – 2.4)	7.8 (4.7 – 20.1) P < 0.001	-	

The data of age and the BALF recovery rate with symmetric distribution are presented as mean ± standard deviation. Sex and the smoking status were evaluated by the chi-square test. The other data show asymmetric distribution, which is presented as a median (interquartile range). P values below each data value were calculated using the Mann–Whitney *U*–test to compare with the IPF group. P values in the rightmost column were calculated using the Kruskal–Wallis test to analyze the distribution of data among the three groups. SP-A, surfactant protein A; SP-D, surfactant protein D; BALF, bronchoalveolar lavage fluid; HS, healthy subjects; IPF, idiopathic pulmonary fibrosis patients; SAR, sarcoidosis patients.

### SP-A and SP-D levels in BALF and serum

The data of SP-A and SP-D levels of each subject group are summarized in Table 1. Serum concentration of SP-A and SP-D in IPF patients [median 84.5 ng/mL (interquartile range 55.5 – 115.8 ng/mL) and 272.0 ng/mL (172.0 – 441.8 ng/mL), respectively] was mostly above the normal upper limit of previous reports [6, 19, 32], and higher than that of SAR patients [33.3 ng/mL (26.1 – 52.0 ng/mL); P < 0.001 and 75.0 ng/mL (48.8 – 97.3 ng/mL); P < 0.001, respectively].

**Figure 1:**
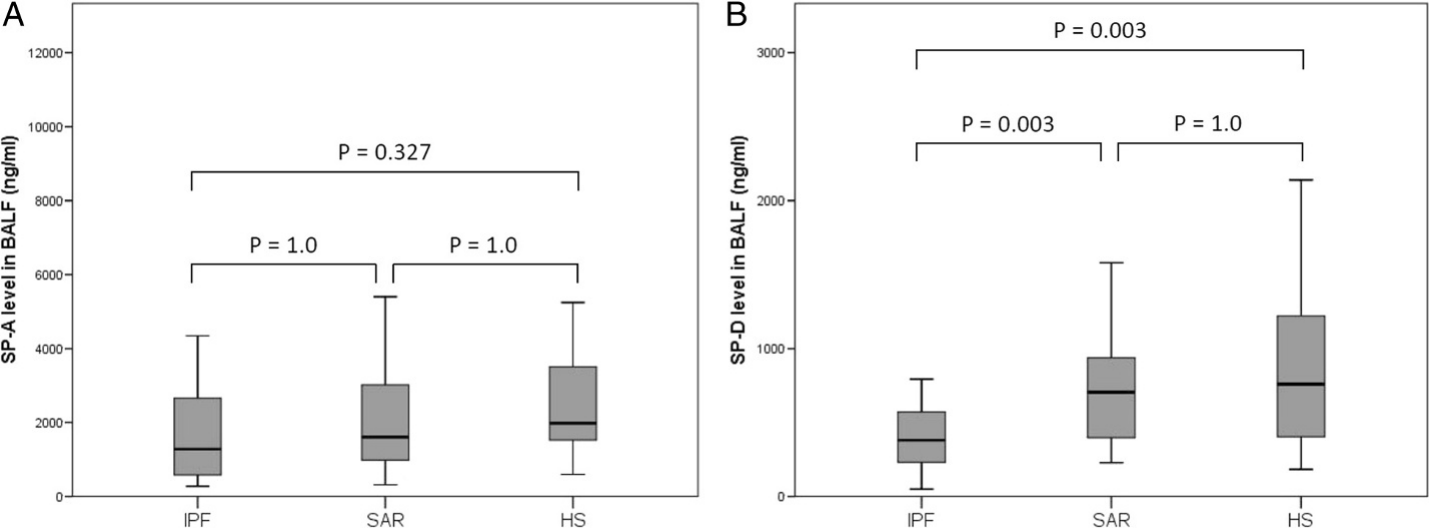
**Surfactant protein (SP)-A and SP-D levels in bronchoalveolar lavage fluid (BALF). (A)** There was no significant difference in SP-A levels between patients and healthy subjects (HS). **(B)** The SP-D levels were significantly lower in idiopathic pulmonary fibrosis (IPF) patients than in healthy subjects (P = 0.003) and sarcoidosis patients (SAR; P = 0.003).

Measurements of SP-A in BALF revealed comparable concentrations between the three groups (P = 0.245): IPF patients (median 1275.0 ng/mL), healthy subjects (median 1978.0 ng/mL), and SAR patients (median 1600.0 ng/mL) (Figure 1A). In contrast, SP-D concentrations in the IPF group (median 379.0 ng/mL) were significantly lower than those in the healthy (median 667.0 ng/mL, P = 0.003) and SAR groups (median 704.5 ng/mL, P = 0.003) (Figure 1B). Because demographic differences were found among groups and some original data of healthy subjects had been lost, using multivariate analysis, we confirmed low BALF SP-D level in the IPF group compared with the SAR group (P = 0.016; Table 2).

**Figure 2:**
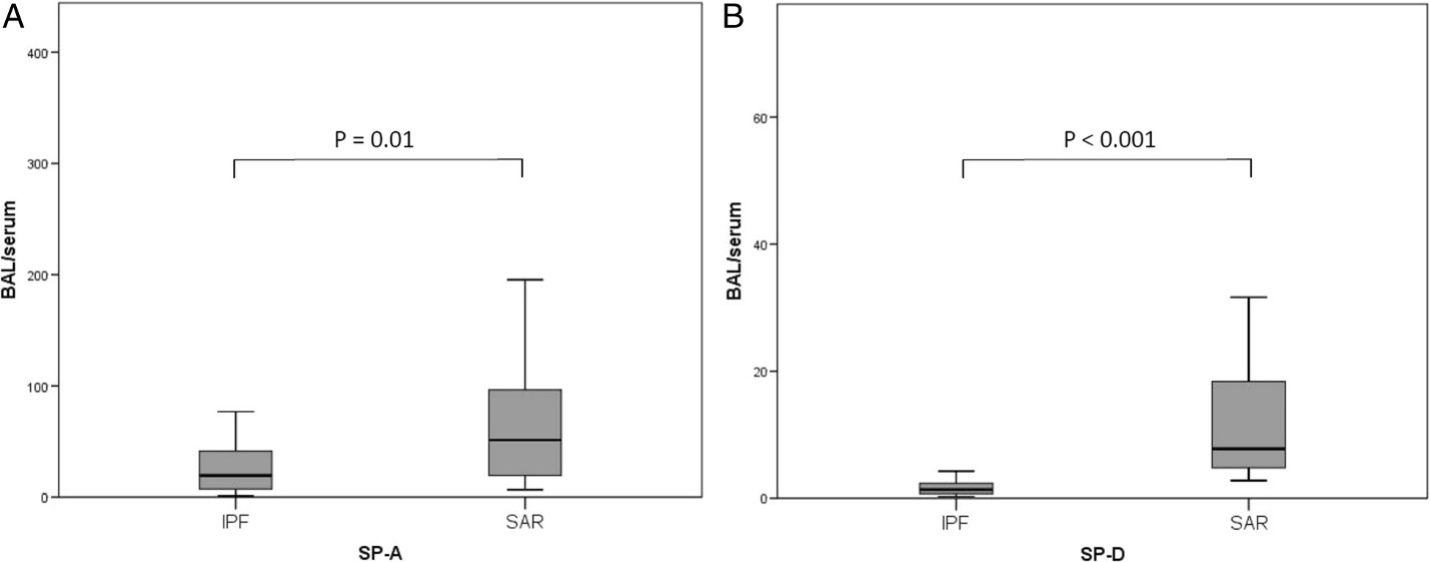
**Bronchoalveolar lavage fluid (BALF)/serum level ratio of (A) SP-A and (B) SP-D.** Concerning both SP-A and SP-D, the ratio was smaller in idiopathic pulmonary fibrosis (IPF) patients than in sarcoidosis (SAR) patients (P = 0.01 and P < 0.001, respectively).

The gaps between BALF and serum level (BALF/serum level ratio) of SP-A and SP-D were median 19.1 (SP-A) and 1.3 (SP-D) in IPF patients, and median 51.2 (SP-A) and 7.8 (SP-D) in SAR patients. These gaps were significantly smaller in IPF patients than in SAR patients (SP-A: P = 0.01 and SP-D: P < 0.001) (Figure 2A, B).

**Figure 3:**
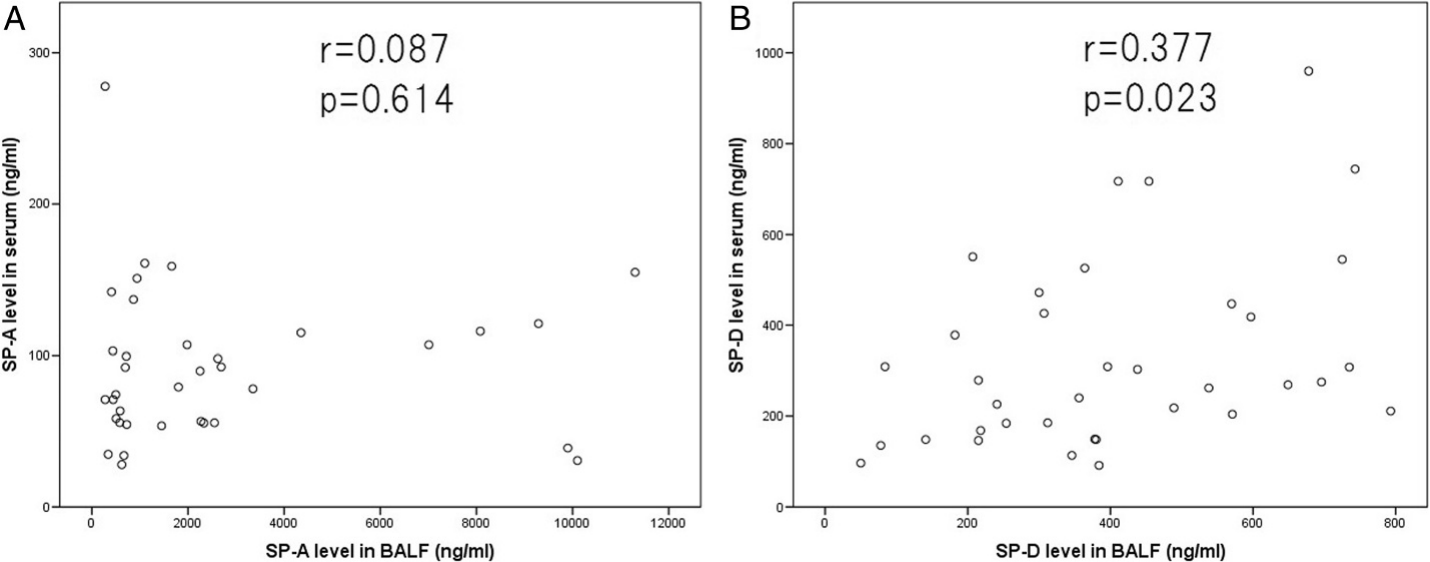
**Surfactant protein levels in serum and bronchoalveolar lavage fluid (BALF) of idiopathic pulmonary fibrosis patients. (A)** There was no correlation between BALF and serum SP-A levels. **(B)** Significant positive correlation between BALF and serum SP-D levels was observed (r = 0.377; P = 0.023).

Spearman’s rank coefficient analysis between BALF and serum concentrations was conducted for each surfactant protein in each disease group. There was no significant correlation for SP-A in the IPF group, but a moderate positive correlation was observed for SP-D in IPF patients (r = 0.377, P = 0.023) (Figures 3A, B). In the SAR group, significant correlation was not observed in both protein concentrations between BALF and sera (data not shown).

**Table 2:** **The differences of each variable between IPF and SAR group**

	Univariate analysis	Multivariate analysis
Sex	P = 0.073	P = 0.056
Age	P < 0.001	P = 0.007
Smoking status	P = 0.528	P = 0.277
BALF SP-A level	P = 0.405	P = 0.082
BALF SP-D level	P = 0.001	P = 0.016

The SP-D level in BALF of IPF patients was significantly decreased compared to SAR patients with univariate and multivariate analysis. IPF, idiopathic pulmonary fibrosis; SAR, sarcoidosis; BALF, bronchoalveolar lavage fluid; SP-A, surfactant protein A; SP-D, surfactant protein D.

### Comparing the hydrophilicity of SP-A and SP-D in BALF

The BALF supernatant fractions from IPF patients centrifuged to remove cellular components (Sup-1) were centrifuged again (33,000 *g*) to generate a hydrophobic precipitate (Ppt) and hydrophilic supernatant (Sup-2). Nearly all the SP-A sedimented in the Ppt (median 97.9%), indicating high hydrophobicity. In contrast, SP-D partitioned into the two phases, but significantly more in Sup-2 (median 52.4%). Finally, nearly all surfactant phospholipids were collected in the Ppt (median 100.0%). Together, these data suggest that hydrophilicity of SP-A is weakened by binding with phospholipids in BALF. The results of centrifugation of BALF are summarized in Table 3.

**Table 3:** **Fragmentation of SP-A and SP-D in the bronchoalveolar lavage fluid of idiopathic pulmonary fibrosis patients**

	Sup-2	Ppt	
SP-A (%)	2.1 (0.9 – 4.4)	97.9 (95.6 – 99.1)	P < 0.001
SP-D (%)	52.4 (48.4 – 79.3)	47.6 (20.7 – 51.6)	P = 0.011
Total Phospholipid (%)	0.0 (0.0 – 8.3)	100.0 (91.7 – 100.0)	P < 0.001

Bronchoalveolar lavage fluid was centrifuged (1,000 *g*) to remove cell components (Sup-1), and Sup-1 was centrifuged again (33,000 *g*) into a supernatant (Sup-2) and precipitate (Ppt). The percentage of fragmentation of SP-A, SP-D and phospholipids in each segment was examined. The data are presented as median (interquartile range). SP-A, surfactant protein A; SP-D, surfactant protein D.

### Immunohistochemistry

**Figure 4:**
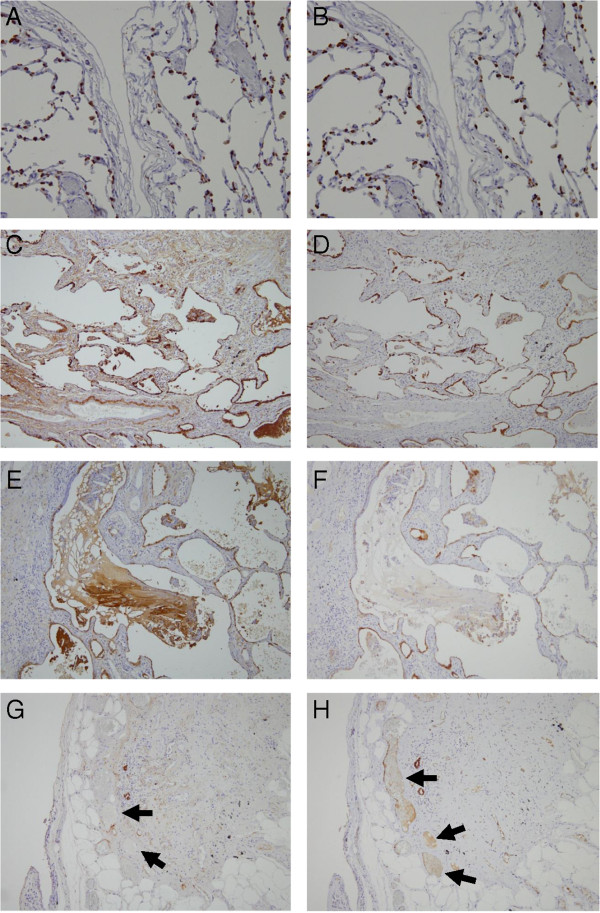
**Immunohistochemistry of control lung tissue and lung biopsies from idiopathic pulmonary fibrosis (IPF) patients. (A-B)** In normal tissue, the cytoplasm of type II alveolar epithelial cells was positive for **(A)** SP-A (brown) and **(B)** SP-D (brown). **(C-D)** In IPF lungs, the type II alveolar epithelial cells were hyperplastic, and strongly positive for **(C)** SP-A (brown) and **(D)** SP-D (brown). **(E-F)** The thick mucus inside honeycomb cysts and ectatic respiratory tracts was highly immunoreactive for **(E)** SP-A but not for **(F)** SP-D. **(G-H)** Inside blood vessels (arrows) were often positive for (h) SP-D but not for (g) SP-A.

**Figure 5:**
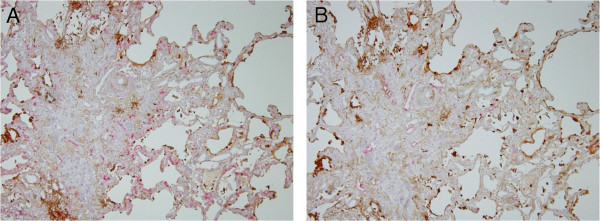
**Immunostaining of blood and lymphatic vessels in lung biopsies from idiopathic pulmonary fibrosis (IPF) patients. (A)** The capillary vessels were extremely developed, especially along the air space (red: CD34, brown: SP-A). **(B)** On the other hand, lymphatic vessels were localized at the center of the stroma (red: D2-40, brown: SP-A).

The tissue distribution of SP-A and SP-D was analyzed by immunostaining of surgical lung biopsies from IPF patients and peripheral noncancerous control lung tissue from lung cancer patients. Type II alveolar epithelial cells, which are known to produce pulmonary surfactant, were positive for both surfactant proteins in normal peripheral lungs (Figures 4A, B) and IPF lungs (Figures 4C, D). In fibrotic lesions where alveoli were severely collapsed, the alveolar space had been covered by regenerated ciliated epithelial cells. These cells were negative for SP-A and SP-D, similar to normal ciliated epithelial cells. The ectatic alveolar space was often filled with thick mucus strongly immunoreactive to SP-A, but weakly immunoreactive to SP-D (Figures 4E, F). Whereas SP-D was often expressed inside blood vessels in the thick stroma, SP-A was always negative (Figure 4G, H). Double immunostaining for CD34 indicated that the capillary vessels adjacent to type II alveolar epithelial cells were extremely hyperplastic (Figure 5A). On the other hand, the lymphatic vessels stained by D2-40 were not located near the alveolar space, but concentrated at the center of the stroma (Figure 5B).

Semi-qualitative analysis of the immunohistochemical distribution comparing SP-A and SP-D is presented in Table 4.

**Table 4:** **Summary of SP-A and SP-D immunolocalization in normal peripheral and IPF lungs**

	SP-A	SP-D
Type II alveolar epithelial cells (in normal lungs)	+	+
Columnar ciliated cells	-	-
Regenerated type II alveolar epithelial cells	++	++
Fibrous parenchyma	- ~ +	- ~ ±
Mucus in honeycomb cysts	+++	- ~ ±
Inside small vessels	-	- ~ +
Elastic fibers in the wall of blood vessels	+	-

Immunostaining intensity: -, (none); ±, (more or less); +, (positive); ++, (more intense); and +++, (very intense). SP-A, surfactant protein A; SP-D, surfactant protein D; IPF, idiopathic pulmonary fibrosis.

## Discussion

It has been reported that serum SP-A and SP-D concentrations are elevated in IPF and other interstitial lung diseases, and that they can predict the progression of respiratory failure and prognosis in IPF [5, 6]. However, there was considerable variability in serum SP-A/SP-D level ratio between patients. As an example for this variability, we presented the difference of serum concentration of these proteins between IPF patients with ground glass opacities (GGO) and patients with consolidation-dominant opacities by computed tomography [5]. Although serum SP-D was elevated in both groups, serum SP-A concentration was high only in patients with GGO. SP-A and SP-D are proteins with high homology in structure and function; however, these observations supported differences in transition efficiency from the alveolar space to the bloodstream.

We previously reported BALF concentrations of SP-D equivalent or lower in IPF patients than in healthy subjects [33]. Likewise, the present study revealed SP-D levels in BALF to be significantly lower in IPF patients than in SAR patients or healthy subjects. Besides, McCormack et al. and Gunther et al. reported significantly lower SP-A concentrations in the BALF of IPF patients than in healthy subjects [33, 34]. In the current study, the SP-A concentration in BALF had a tendency to decrease among IPF patients compared with healthy subjects, but the difference was not significant. Although the studies on the two proteins of BALF show the same directionality, we find different amplitudes in their levels between IPF and normal lungs. The reasons for these differences are unclear. One potential explanation is the ATS/ERS(/JRS/ALAT) guideline for idiopathic pulmonary fibrosis was revised in 2000 [35] and 2010 [25] and the current disease group may differ from those in previous studies.

According to the current study, the BALF/serum level ratio of SP-A was reduced from 51:1 in the sarcoidosis group to 19:1 in the IPF group. In contrast, IPF caused a 6-fold decrease in BALF/serum level ratio for SP-D, with 7.8:1 in the sarcoidosis group compared with 1.3:1 in the IPF group. These data suggest that IPF caused a preferential leakage of SP-D from the alveolar space into the bloodstream. Moreover, in this study, the SP-D concentrations in sera were correlated with those in BALF, but such a correlation was not found for SP-A. This result may be due to the higher permeability of SP-D, implying that not only BALF concentration but also serum concentration can be easily altered according to the amount of secretion of SP-D from productive cells. Mogulkoc et al. reported that the clearance of inhaled technetium 99 m-labelled diethylenetriamine penta-acetic acid (^99m^Tc-DTPA) aerosol was exacerbated among IPF patients and suggested that the migration of substances from the alveolar space into the bloodstream was accelerated [36]. One of the reasons for this high permeability may be the impairment of the basement membrane in IPF lungs where alveoli were collapsed and replaced with fibrotic interstitium [37, 38]. This impairment of the basement membrane seems to promote the transition of SP-A and SP-D into the bloodstream.

The present study revealed dissociation between SP-A and SP-D in IPF lungs, suggesting that different protein–lipid interactions may affect their leakage into the bloodstream. Most of the SP-A found in the alveolar space is bound to DPPC, which is the main component of pulmonary surfactant [9]. In contrast, SP-D remains in a lipid-free state in the alveolar space [11, 39], which could facilitate leakage from airspace into bloodstream. Accordingly, the hydrophilicity experiments conducted with BALF from IPF patients showed that nearly all SP-A was sedimented in the Ppt after high-speed centrifugation, consistent with SP-A-DPPC complexing. In contrast, SP-D partitioned significantly in the hydrophobic (Ppt) and hydrophilic (Sup-2) fractions. Accordingly, SP-D (but not SP-A) was often immunolocalized inside blood vessels in the fibrotic parenchyma. In contrast, SP-A (but not SP-D) remained confined to the thick mucus-filling the alveolar space. Because this mucus abundantly contains pulmonary surfactant, SP-A binding with phospholipids are considered to be profusely present there. These findings are consistent with the preferential leakage of SP-D into the bloodstream.

CD34 is known as a marker of vascular endothelium [30, 40]. Ebina et al. reported that relatively small vascular endothelial cells, like capillaries, are more intensively stained by CD34 antibodies than proximal vessels [41]. However, D2-40 is a marker of lymphatic endothelium frequently used to detect the presence of lymphatic invasion of malignancies [31]. In our study, CD34 detected remarkable hyperplasia of the capillaries in the thickened stroma, particularly near the alveolar space, in IPF lungs. These capillaries were close to the regenerated type II alveolar epithelial cells that where SP-A and SP-D positive. Hisata et al. suggested that surfactant proteins may be transferred from type II cells to endothelial cells of adjacent capillaries in IPF lungs [42]. On the other hand, we showed that lymphatic ducts are concentrated in the central portion of fibrotic interstitium, away from the alveolar space and type II cells. According to this positional relationship, surfactant proteins would leak into the bloodstream directly via capillaries, and not through lymphatic ducts.

This study has some limitations. We should interpret the concentrations in BALF not only from the viewpoint of the leakage and clearance but also from that of the alterations in the production amount in IPF lungs. Because both proteins have the important function to regulate the inflammatory cell response, their productions may be altered in IPF lungs. Further examinations are required regarding these issues. We used the data of BALF from healthy volunteers who were investigated in our previous study [26]. Because SP-A and SP-D levels in sera had not been evaluated, it was not possible to obtain correlations between BALF and serum levels in healthy subjects. In addition, there was a disparity between demographic data of these healthy subjects and those of IPF patients. Unfortunately, because some original data from healthy subjects had been lost, we could not include them in multivariate analysis. We accordingly compared the data from IPF patients with those from sarcoidosis patients as a disease control. Demographic data from the patients, age, smoking status, infection, and nutrition status might affect the permeability of alveolocapillary barrier. Although we found no significant difference in smoking history or BALF recovery between the IPF and SAR groups and any infection or nutrition problems in these two groups, the age bias remained. We confirmed a low BALF SP-D level in the IPF group using multivariate analysis. Because the histopathological pattern of IPF shows temporal and spatial heterogeneity, the site of sampling may play an important role. We collected BALF from the right middle lobe or lingula, and obtained histologies from the most lesioned areas. These contradictory sites may have affected the results of our study. The new BAL guideline published by ATS in 2012 [43] recommends that the BAL target site should be chosen on the basis of an high-resolution computed tomography performed before the procedure in preference to a “traditional BAL site” such as the right middle lobe or lingula. Further studies should be conducted in accordance with the new guideline. With reference to the immunohistochemical study, we should mention the possibility that SP-D might be more easily lost from the bronchoalveolar space during immunohistochemistry process because of its hydrophilicity. However we identified strong immunoreactivity to SP-D in normal/hyperplastic type II alveolar epithelial cells with the same degree to SP-A and believe that this technical bias is small.

The histopathological features of IPF indicate a heterogeneous appearance. Honeycombing, in which type II cells are replaced with regenerated ciliated epithelial cells, is a finding that represents the end stage of fibrotic change, and this finding seldom regresses. In contrast, alveolitis is a potentially reversible and treatable parenchymal abnormality. In such a lesion, type II alveolar epithelial cells are extremely hyperplastic. These variable features may reflect dynamic differences between SP-A and SP-D because of their hyprophilicity. If serum SP-D levels mirror the severities of moderate and reversible damage to the epithelium due to their ready leakage into the bloodstream, these levels can be used as a biomarker to predict therapeutic response. We need to assess the utilities of these biomarkers with regard to the therapeutic effects of drugs such as antifibrotic agents. Currently, KL-6, MMP-7 and CCL-18 have been also reported as blood biomarkers of IPF [20–22]. Whereas all of them are elevated in the serum of IPF patients, they may accumulate for different reasons. Song et al. suggested that multiple markers may be required to improve the prediction of prognosis and disease progression in IPF patients [21]. We believe that simultaneous measurements of several biomarkers with unique characteristics will provide a more sensitive readout of the temporal changes in pathological profile of IPF.

## Conclusions

Although SP-A and SP-D are serum biomarkers of IPF having high homology, the current study suggests that the difference in hydrophilicity of the two proteins could be the cause of their difference in migration from the air space into the bloodstream. Because SP-D leaks into the circulation more easily, serum levels of SP-D may reflect pathological changes of the disease more sharply than those of SP-A.

### Consent

Written informed consent was obtained from the patient for the publication of this report and any accompanying images.

## Abbreviations

SP: Surfactant protein
IPF: Idiopathic pulmonary fibrosis
BALF: Bronchoalveolar lavage fluid
SAR: Sarcoidosis
Sup: Supernatant
Ppt: Precipitate
DPPC: Dipalmitoyl phosphatidylcholine
FVC: Forced vital capacity
D_LCO_: Diffusing capacity of the lung for carbon monoxide
KL-6: Krebs von den Lungen-6
MMP-7: Matrix metalloproteinase-7
CCL18: Chemokine (C-C motif) ligand 18
BAL: Bronchoalveolar lavage
ATS: American Thoracic Society
ERS: European Respiratory Society
JRS: Japanese Respiratory Society
ALAT: Latin American Thoracic Association
ELISA: Enzyme linked immunosorbent assay
GGO: Ground glass opacities
Tc-DTPA: Technetium 99 m-labelled diethylenetriamine penta-acetic acid.
